# Perioperative management of gastrointestinal surgery in a resource-limited hospital in Niger: Cross-sectional study

**DOI:** 10.1016/j.amsu.2020.03.009

**Published:** 2020-04-13

**Authors:** Harissou Adamou, Ibrahim Amadou Magagi, Ousseini Adakal, Mahamadou Doutchi, Oumarou Habou, Mamane Boukari, Lassey James Didier, Rachid Sani

**Affiliations:** a Department of Surgery and Surgical Specialties - Zinder National Hospital, Faculty of Health Sciences, University of Zinder, Niger; b Surgery Department, Maradi Hospital Center, Faculty of Health Sciences, University of Maradi, Niger; c Infectiology Department - Zinder National Hospital, Faculty of Health Sciences, University of Zinder, Niger; d Department of Surgery and Surgical Specialties, Faculty of Health Sciences, University of Niamey, Niger

**Keywords:** Perioperative, Management, Gastrointestinal surgery, Complications, Quality of care

## Abstract

**Background:**

Perioperative management in digestive surgery is a challenge in sub-Saharan Africa. Objective: To describe the process and outcomes of perioperative management in gastrointestinal surgery.

**Materials and methods:**

This was a single center cross-sectional study over a 4-month period from June 1 to September 30, 2017, in a Nigerien hospital (West Africa). This study included caregivers and patients operated on gastrointestinal surgery.

**Results:**

We collected data for 56 caregivers and 253 patients underwent gastrointestinal surgery. The average age of caregivers was 38.6 ± 8.7. The median length of professional practice was 9 years. Almost 52% of caregivers (n = 29) did not know the standards of perioperative care. The median age of patients was 24 years, and male gender constituted 70% of cases (n = 177) with a sex ratio of 2.32. Patients came from rural areas in 78.2% (n = 198). Emergency surgery accounted for 60% (n = 152). The most surgical procedure was digestive ostomies performed in 28.9% (n = 73), followed by hernia repair and appendectomy in 24.5% (n = 62) and 13.9% (n = 35) respectively. The postoperative course was complicated in 28.1% (n = 71) among which 13 deaths. In the group of caregivers, the poor practice of perioperative management was associated with poor professional qualification, insufficient equipment, insufficient motivation (p < 0.05). The ASA3&ASA4 score, undernutrition, emergency surgery, poor postoperative monitoring, and poor psychological preparation were associated with complicated postoperative outcomes (p < 0.05).

**Conclusion:**

The inadequacy of the technical platform and the lack of continuous training for healthcare staff represented the main dysfunctions of our hospital. The risk factors for complications found in this study need appropriate perioperative management to improve prognosis in gastrointestinal surgery.

## Highligth

• Perioperative care in gastrointestinal surgery in a challenge in Sub-Saharan countries.
• This setting is characterised by the difficulty of geographical access to hospital, lack of equipment.
• Risk factors for complications are multifactorial.
• This study can be a draft for improvement the quality of care in low resource settings.

## 1 Introduction

Surgery, an important component of health systems that has been neglected by global public health policies, has undergone a remarkable evolution in recent years [[1], [2], [3], [4]]. In 2004 and 2012, 234 million and 312 million major surgical procedures were performed respectively. Only 6% are carried out in the poorest countries, which account for more than a third of the world's population [2,3]. Gastrointestinal surgical diseases are common affections, which cause considerable morbidity and mortality, particularly in sub-Saharan Africa [1,[3], [4], [5], [6], [7], [8], [9]]. These complications are indicators to monitor the quality of the surgical care provided [4,5]. In this setting of lack of resources, several factors influence the quality of perioperative management such as lack of qualified caregivers, diagnostic delay, lack of communication, insufficient equipment and consumables [1,[3], [4], [5], [6], [7], [8], [9], [10]]. It has been shown that even for low-risk patients, postoperative mortality in Africa is twice as high the global average [8]. Currently, surgeons are increasingly concerned with perioperative nonsurgical aspects. Indeed, the success of surgical management is not limited to the mere surgical procedure [[11], [12], [13]]. This management must be ensured whole, patient-centered approach that includes all stakeholders taking into account the technical platform, the overall perioperative state [12,13]. The implementation of multiple perioperative actions allows the patient to be treated effectively, efficiently and safely [2,4,5,[11], [12], [13]]. In our context, perioperative care in gastrointestinal surgery is dysfunctional, which explains this high morbidity and mortality [9,10]. The objective of this study was to describe the process and outcomes of perioperative management in gastrointestinal surgery in a resource-limited hospital.

## 2 Methods

This study had been registered in accordance with the declaration of Helsinki at the Research 2019. The registration number is researchregistry5224. The ethical approval was obtained from the relevant hospital and university authorities. This work has been reported in line with the STROCSS criteria [14].

This was a single center cross-sectional study over a 4-month period from June 1 to September 30, 2017, in a southeastern hospital of Niger Republic in Zinder area. This is a 800-bed tertiary public hospital serving Zinder area which had about 4.3 million inhabitants in 2017 and receives references from three neighboring regions of the country (Agadez, Diffa and Maradi). Niger Republic is a West African country ranked in 2018 189th out of 189 countries according to the United Nations in terms of Human Development Index (0.354) with a multidimensional poverty index of 0.154 and an average income per capita per day of less than one dollar. A health system that is still fragile, and access to quality care remains a challenge for the majority of the population. The assessment of the perioperative care structure was performed and, at the same time, the study concerned patients and caregivers. In this study caregivers are defined as all the health agents involved in perioperative care (medical doctors, surgeons, nurses, anesthesia technicians, surgical assistant, social worker).

The study population included patients undergoing gastrointestinal surgery (emergency or elective surgery) and the caregivers who work in the gastrointestinal surgery department. Caregivers absent during the study period, those who did not agree to participate in the survey. Non-consenting patients and patients with gastrointestinal diseases who were not operated on were excluded from the study. General, regional or local anesthesia was used to perform surgery. Surgical procedure was performed by a team of senior surgeons and physicians in training for surgery in a district hospital.

At the inventory of surgical services with 3 kinds of performance judgments (good, fair and bad):

• Poor: When the situation is deemed inadequate with an Absolute Need of Improvement (ANI);
• Fair: When the situation is considered acceptable, but deserves to be Improved (I);
• Good: When the situation judged is good, Good performance (G).

The perioperative course of the patients was analyzed. Semi-open survey sheets for caregivers and patients have been established. In order to measure the comprehension and easy interpretation of these sheets; a pre-test was conducted in 8 agents and 10 patients. Inpatient and operating room registers were used to supplement information on patients' perioperative management. Data collected included many variables for caregivers: gender, age, length of time in the function, level of knowledge on perioperative care, effectiveness of continuing education, qualification: Certified Nurse (CN), State Registered Nurse (SRN), Senior Nurse (SN) or Physician. For the patients, the variables studied were: origin, age, sex, the marital status, the anthropometric parameters, indications of surgery, the gestures, the complications and the discharge mode. Digestive stomas were fitted with plastic bags that could easily be changed by the patient or his or her family and friends. The general condition was evaluated by the ASA (American Society of Anesthesiologists) score [15]. The clinical assessment of nutritional status was routinely based on the body mass index (BMI) expressed in Kg/m^2^. The undernutrition is evoked for a BMI value < 18.5 kg/m^2^, in adults aged 20 years and over. For patients aged 5 to 19 and those under 5 years of age, the 2007 WHO sheets were used to determine nutritional status (WHO Z-score) [16]. The degree of surgical contamination was distributed according to Altemeir's classification [17] and the complications (30-day post morbidity/mortality) were categorized according to Clavien-Dindo [18].

The data collected was captured and analyzed using Excel and Epi-Info^7TMCDC^ software. Quantitative variables were expressed as mean ± standard or median deviation with interquartile range (IQR: Q1-Q3) and the Kruskal Wallis test was used to make associations. Qualitative variables were represented as percentages or number and associations were made with Pearson's Chi-square or Fisher's tests. A significance level of 5% was used for all tests.

## 3 Results

### 3.1 Inventory of the structure

The inventory allowed us to make a synthesis by carrying 3 kinds of judgments: bad, fair, and Good is summarized in Table 1. It is clear that the dysfunctions noted required is an absolute need to improve the technical platform. These dysfunctions result in a postponement of 27.3% (n = 38) of scheduled surgical procedures for gastrointestinal diseases.

**Table 1:** overview of the state of the surgical services.

Topics	ANI	I	G
1. Care circuit	X		
2. Materials and Equipements	X		
3. Drugs and Consumables	X		
4. Laboratory tests		X	
5. Cleanliness and privacy of the premises (hospital rooms and toilets)	X		
6. Data recording media		X	
7. Availability of caregivers		X	

Poor: When the situation is deemed inadequate with an Absolute Necessity of Improvement (**ANI**).

Fair: When the situation is considered acceptable, but deserves to be Improved (**I**).

Good: When the situation judged is good, Good performance (**G**).

### 3.2 Characteristics of caregivers

**Fig. 1:**
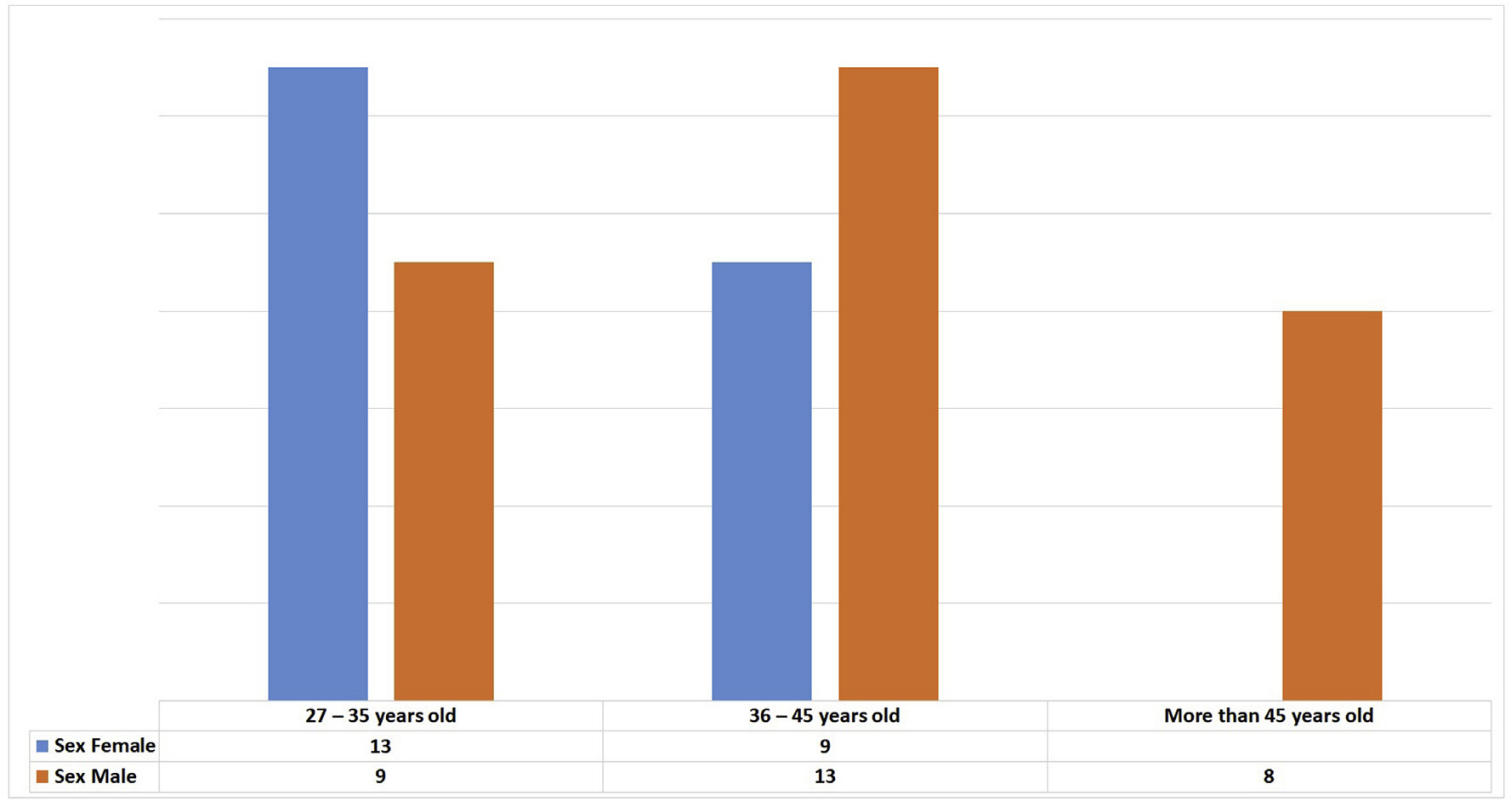
Distribution Caregivers -age and sex.

During the study period, we interviewed 56 caregivers involved in the perioperative management. The age of caregivers ranged from 27 to 58 years with an average of 38.6 ± 8.7 years. The male sex predominated with 60.7% (n = 34), a sex ratio of 1.54. Fig. 1 shows the distribution of caregivers by age group and gender. By marital status, married, single, divorced and widowed were respectively 76.8% (n = 43), 17.8% (n = 10), 3.6% (n = 2) and 1, 8% (n = 1).

According to the qualifications, 89.3% (n = 50) were non-physicians and 10.7% (n = 6) were physicians. State registered nurse (n = 28) and certified nurses (n = 12) accounted for 80% (n = 40) of all nurses, while Senior Nurse (SN) comprised 20% (n = 10). The duration of professional practice at the HNZ ranged from 3 to 35 years with a median of 9 years (IQI: 7–13.5 years). Almost 52% of caregivers (n = 29) did not know the definition and content of perioperative care. The main difficulties encountered in perioperative management were: the lack of training in 82.1% (n = 46), the inadequacy of adapted equipment in 66.1% (n = 37). Lack of motivation was noted in 58.9% of agents (n = 33) and overwork was reported by 42.9% of providers (n = 24). Correct practice of perioperative management was statistically associated with occupational qualification (OR = 8.04 [1.95–33.08], p = 0.0026). The poor practice of care was related to insufficient equipment (OR = 3.55 [1.10–11.50], p = 0.030), lack of motivation (OR = 4.57 [1.45–14.69], p = 0.007) and overwork (OR = 4.04 [1.30–12.58], p = 0.013). However, the length of professional exercise of non-physician caregivers was not statistically associated with the knowledge and application of standard norms in the perioperative PEC (Kruskal-Wallis H = 2.96, p = 0853).

### 3.3 Characteristics of patients

**Fig. 2:**
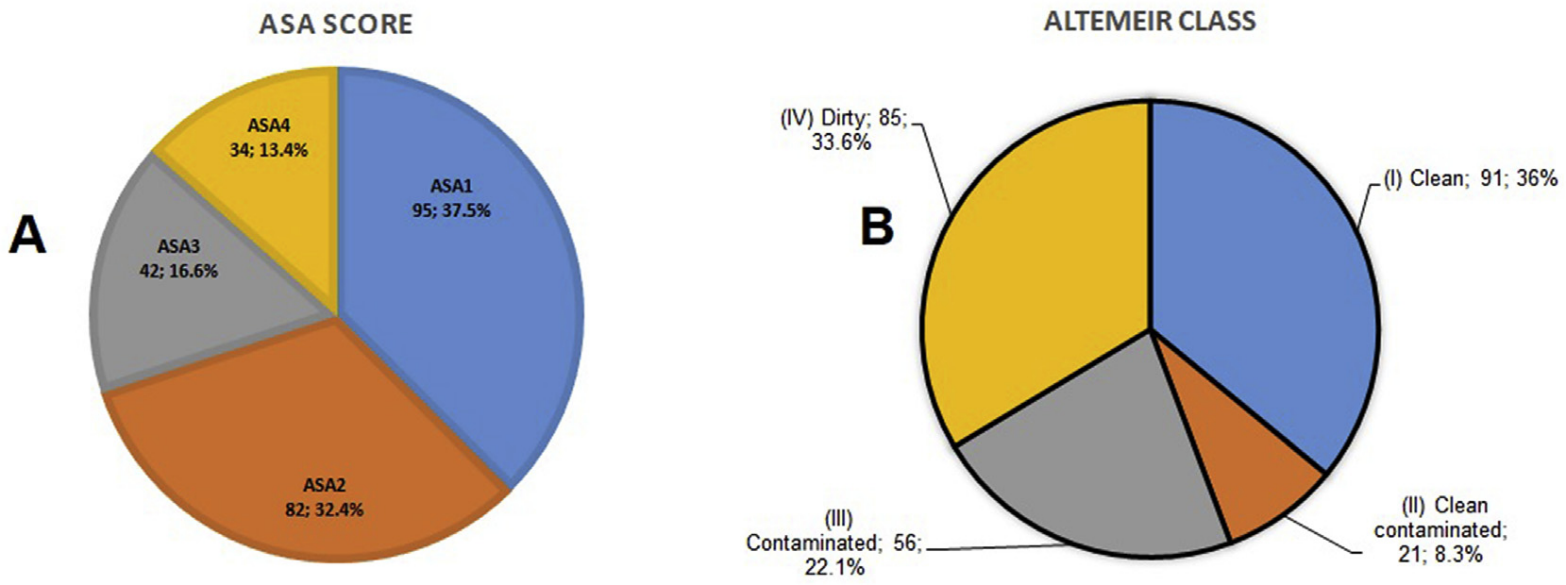
ASA score & altemeir class.

A total of 481 operative procedures performed, 253 patients underwent gastrointestinal surgery (52.6%). Patients were from rural areas with difficulty of geographical access to hospital in 78.2% (n = 198). The age of our patients ranged from 0 to 93 years with a median age of 24 years (IQR: 10–45 years). The pediatric population aged 0 to 15 represented 39.9% (n = 101). The male sex accounted for 69.96% (n = 177) with a sex ratio of 2.32. Table 2 shows us the distribution of patients by age and sex. The distribution of patients by admission mode shows that direct consultations, evacuations and referrals accounted respectively for 48.6% (n = 123), 27.7% (n = 70) and 23.7% (n = 60). Patients were classified ASA1 in 37.5% (n = 95) and ASA2 in 32.4% (n = 82). In this series, class III (contaminated) and IV (dirty) of the Altemeir classification accounted for 55.7% (n = 141), Fig. 2.

**Table 2:** Distribution of patients by age and sex.

Age group (years)	Gender		Total (%)
	Female	Male	
0–15	34	67	101 (39.9)
16–30	14	41	55 (21.7)
31–45	13	28	41 (16.2)
46–60	10	26	36 (14.2)
More than 60	5	15	20 (7.9)
**Total (%)**	**76 (30.04)**	**177 (69.96)**	**253 (100)**

Surgical emergency was required in 60% (n = 152) while elective surgery was performed in 40% of cases (n = 101). Emergency surgery was performed within 1–8 h of admission in 80.9% (123/152). General anesthesia was used in 75.9% (n = 192) and locoregional anesthesia in 24.1% (n = 61). Laparotomy was the main surgical approach performed in 74.7% (n = 189). Digestive ostomies were performed in 28.9% (n = 73), followed by hernia repair and appendectomy in 24.5% (n = 62) and 13.8% (n = 35). Table 3 gives us the distribution of surgical procedures and operative indications.

**Table 3:** Distribution of surgical procedure and indications (n = 253).

Indications		Number (%)
Emergency surgery: 60% (n = 152)	Peritonitis	84 (33.2)
	Mechanical intestinal obstruction	31 (12.2)
	Appendicitis	24 (9.5)
	Abdominal trauma	13 (5.1)
Elective surgery: 40% (n = 101)	Hernia	62 (24.5)
	Incisional hernia	13 (5.1)
	RDC*	13 (5.1)
	Tumeur colorectale	11 (4.3)
	Fistule anale	2 (0.8)
**Surgical procedures**		
Laparotomy (n = 189)	Ileostomies	58 (22.9)
	Colostomies	15 (5.9)
	Ileal resection and anastomosis	25 (9.9)
	Ileal suture	11 (4.3)
	Appendectomy	35 (13.8)
	Right hemicolectomy	5 (2)
	Left colectomy	8 (3.2)
	Splenectomy	5 (2)
	Incisional hernia repair,	13 (5.1)
	*Others	14 (5.5)
Hernia repair (n = 62)		62 (24.5)
Fistulectomy (2)		2 (0.8)

*RDC: Restoration of digestive continuity.

*Others: Haemostasis (n = 5), gastric sutures (n = 4), peritoneal toillette (n = 4).

In this series, the surgical safety checklist as recommended by WHO was not completed in the operating room. More than 25% (n = 65) of cases were malnourished, but only 18.5% (n = 12) of them had received a perioperative nutritional supply. The dysfunctions in perioperative physical and psychological management are listed in Table 4. Perioperative whole blood transfusion was done in 33.6% of patients (n = 85), including 52 cases of peritonitis, 12 cases of abdominal trauma, 11 cases of intestinal obstruction and 11 cases of colorectal tumors.

**Table 4:** The different items evaluated perioperatively.

Perioperative items evaluated	Number	(%)
Perioperative Nutrition with RUTF^a^	12/65	18.5
Psychological preparation	66/253	26.1
Preoperative shaving	16/75	21.3
Preoperative shower	75/253	29.6
Preoperative fast (>4 h)	208/253	82.2
Checking the operative kit	211/253	83.4
Thromboprophylaxis	22/152	14.5
PAC^b^	253/253	100
Correction of anemia	85/85	100
Antibioprophylaxis	112/112	100
Antibiotic therapy	130/141	92.2
Intraoperative analgesia	211/253	83.4
OpR^c^	198/253	78.3
Good postoperative monitoring	145/253	57.3
Postoperative analgesia	245/253	96.8
Good hygiene and comfort care	11/253	4.3
Bladder drainage	130/170	76.5
Nasogastric tube	121/150	80.7

a RUTF: Ready-to-use therapeutic food used pre or postoperatively by enteral route.

b PAC: Pre-anesthetic consultation.

c OpR: Operative report.

The postoperative outcomes were uneventful in 71.9% (n = 182) of cases. Complications were recorded in 28.1% of cases (n = 71) including 13 deaths (5.1%). Emergency surgery accounted for 83.3% (11/13) of death. The two deaths recorded in elective surgery were tumors. The complications distributed according to Clavien-Dindo are shown in Table 5.

**Table 5:** Distribution of patients by postoperative course.

Postoperative course		Number (%)
Uneventful		182 (71.9)
Complications	(Clavien-Dindo grade)	71 (28.1)
Grade I	Superficial surgical site infection	31
Grade II	Surgical site infection + anemia	10
Grade III	Deep surgical site infection	8
	Evisceration	4
Grade IV	Postoperative peritonitis with renal failure	5
Grade V	Death	13 (5.1)

Among patients with ostomies, 15.1% (11/73) had psychological complications related to loss of self-esteem and body image (n = 4), anxiety (n = 5) and even depression (n = 2). The ASA3 and ASA4 scores, undernutrition, emergency surgery, poor postoperative monitoring, and psychological unpreparedness were all associated with complicated postoperative outcomes (p < 0.05). Association between perioperative factors and postoperative complications are shown in Table 6.

**Table 6:** Associations of care-related factors with postoperative complications.

Prognostic factors		Complications	OR_IC95%_	P value
ASA score	ASA1+ASA2	41/177		
	ASA3+ASA4	30/76	2.16 [1.21–3.85]	0.0081
Nutritional status	Normal-nourished	41/188		
	Malnourished	30/65	3.07 [1.68–5.58]	0.0001
Anemia	No	39/168		
	Yes	32/85	1.99 [1.33–3.51]	0.0158
Type of surgery	Elective	19/101		
	Emergency	52/152	2.24 [1.23–4.09]	0.0077
Altemeier class	C + CC*	18/112		
	Contaminated + dirty	53/141	3.14 [1.71–5.78]	0.0001
Postoperative monitoring	Good	32/145		
	Wrong	39/108	1.99 [1.14–3.47]	0.0141
Psychological preparation for ostomy	Yes	2/188		
	No	9/66	0.07 [0.14–0.32]	0.0001

***C + CC =****clean + clean-contaminated*.

***OR***_***IC95%:***_*Odds ratio with a 95% confidence interval. The reference value of the OR is 1*.

## 4 Discussion

This study, carried out in ZNH, allowed to diagnose the material dysfunctions, identify the difficulties of healthcare workers in perioperative management of gastrointestinal surgery. Indeed, the insufficiency of materials, drugs and consumables requires an absolute improvement. Strengthening the capacity of surgical services in hospitals is essential to reduce the burden of surgical gastrointestinal diseases [1,[6], [7], [8],[19], [20], [21], [22]]. This gloomy situation is already described in many low-income countries [[2], [3], [4], [5], [6], [7], [8],^21^]. On the other hand, the study founded the associated perioperative factors of morbidity and mortality. The occurrence of these complications is an important element in assessing the quality of care. In sub-Saharan Africa, gastrointestinal surgery is a challenge and lead to higher morbidity and mortality [[2], [3], [4], [5], [6], [7], [8], [9],^19^]. Several elements in this study show insufficient motivation of caregivers; yet, it remains irrefutable that the motivation of the staff contributes to an organization's performance [[2], [3], [4], [5], [6], [7], [8],^20^,^21^]. At this level, we note a lack of will on the part of political decision-makers and the government to allocate more resources in the health field. Indeed, improving our hospitals first requires strengthening the technical platform in terms of human resources and equipment. There is also a need for a more equitable distribution and allocation of health workers, taking into account the number of the population and their health needs. Currently, the concept of quality of care in surgery concerns both developed and developing countries [[2], [3], [4],^8^,[23], [24], [25], [26]]. Since 2008, there has been renewed interest in the scientific community in quality of care, patient-centered care, surgical safety, and access to essential and emergency care in surgery [1,5,6,21].

The lack of continuing training and the educational level of caregivers were statistically associated with poor perioperative care practice (P < 0.05) in our study. However, seniority in professional practice was not associated with good perioperative management (p > 0.05). This situation can be explained in our institution, on the one hand, by the large number of certified nurses (short training) and, on the other hand, by the lack of capacity building leading to adopt a bad routine in the practice of care. In their study, Gordon et al. [26] demonstrated that long hospital experience is associated with good practice and a significant decrease in hospital mortality from digestive surgery. Beyond the level of training and compliance with standards of care, the surgical management of gastrointestinal diseases must be carried out in a global approach centered on the patient in its psychological and physical component, taking into account its overall perioperative condition [[11], [12], [13],^22^,[27], [28], [29], [30]]. This includes the involvement of multidisciplinary actors in a good organization [12,13,23,31]. The implementation of multiple perioperative actions allows the patient to be treated effectively, efficiently and safely; this reduces the length of stay, the mortality and the cost of the care [2,3,12,14]. Currently, in sub-Saharan Africa, even when patients are in good general status and have a low operative risk, complications and postoperative deaths are higher than in the rest of the world [6,8,9,32].

Most studies in sub-Saharan Africa, patients are young, but often admitted late in an emergency setting [1,4,[6], [7], [8], [9], [10],^20^]. In this study, the median age of our patients was 20 years and 60% of the interventions were emergency. This shows the delay in consultation or diagnosis already reported in previous publications [9,10]. This delay is multifactorial cause; sometimes related to the socio-economic status of patients, the difficulty of accessibility or the failure of peripheral care facilities [[1], [2], [3], [4],[6], [7], [8], [9], [10]]. The majority of our patients came from disadvantaged rural areas. Lachand [6] reported in this regard: *"The insufficiency of the surgical care supply is even more marked for the rural populations, which are still in the majority. Because it is not enough to know the existence of a high-performance hospital, it is also necessary to be able to access it quickly"* [6]. The majority of our patients were from rural areas (79.1%) and over 94% of gastrointestinal surgical conditions supported in this study corresponds to "essential" surgery and emergency surgery. These conditions can be prevented or managed before complications occur [6,8].

Nowadays, nonsurgical perioperative aspects are of increasing interest to surgeons as well as surgical procedures [11,22,30]. In this study, the perioperative preparation was not optimal and many recommendations are not respected faults of equipment, consumables, lack of knowledge or organization. Admittedly, inputs such as parenteral nutrition, immuno-nutrition, products of mechanical preparation of the colon are often missing in our context, but the checklist, other elements of physical and psychological preparation can be used to reduce complications. Numerous studies have shown the importance of perioperative care and the use of the checklist to reduce the morbidity and mortality of digestive surgery [[3], [4], [5], [6], [7], [8],^13^,^19^]. Adherence to the essential objectives of surgical safety checklist recommended by WHO remains a challenge in our hospital. In this study, vulnerable or avoidable problems such as poor nutritional preparation, emergency or septicity of surgery, poor postoperative monitoring by the caregivers were statistically associated with the occurrence of complications (p < 0.05). Assessment of nutritional status and correction of undernutrition is essential before any major surgery [12,13,28,32]. The undernutrition and anemia are frequently diagnosed before gastrointestinal surgery and are associated with a significant risk of postoperative complications [13,28,32]. More than 33% of our patients had received a blood transfusion for chronic and/or acute anemia. The presence of this was associated with the occurrence of complications (p < 0.05). In our settings, poor psychological preparation of patients is provided by unqualified staff. This perioperative management is not only concerned with surgical procedures and traditional nursing [11,22,28,31,33]. Other aspects, such as the consideration of psychological aspects, are of paramount importance [22,23]. In our study, psychological complications were associated with poor preoperative preparation of patients with digestive ostomy. The majority of these stress, anxiety, distress, depression, loss of body image [[27], [28], [29], [30]]. These psychological repercussions could be reduced by psychological support [28,33]. All these elements of perioperative management demonstrate that the ultimate goal is not only to ensure patient survival [8,11,24,27,33]. All caregivers should provide patient-centered care responsibly, effectively and efficiently. This will preserve the physical, psychosocial and cognitive integrity, but also a satisfaction of patient [13,24,28,30,33]. The quality of care providers’ communication with patients is an important element of patient satisfaction in the perioperative care process [24,33]. The introduction of a quality assurance system in our hospital is an approach that would lead to improved perioperative management. For this change too successful, there must also be the availability of resources, a reorganization of the perioperative follow-up as recommended by numerous studies and regular follow-up to provide solutions to everyday problems [[1], [2], [3], [4],[6], [7], [8],^20^,^23^].

Limitations: Quality assessment in a hospital department is a complex task. This implies that this work has limitations because it does not take into account other important elements covering the structure, the process and outcomes of gastrointestinal surgical diseases. This was a cross-sectional 4-month study that included only 253 patients and 56 caregivers. Other aspects of the hospital's process and structure have not been detailed. However the results obtained in this study have been discussed with literature data and our findings can be used to improve the management. This work is a contribution to the perioperative management of patients in our hospital with resources limited.

## 5 Conclusion

Perioperative care in gastrointestinal surgery involves evaluating the structure, process and outcomes of management. This study allowed us to identify, on the one hand, the lack of materials and consumables, the lack of continous training and motivation, the lack of knowledge of standards. On the other hand, perioperative information has made it possible to identify several risk factors for complications such as undernutrition, anemia, lack of psychological preparation, grade of contamination. This study can be a draft that would guide our hospital towards a improvement approach of the quality of care. Successful change also requires the availability of resources, the detection and correction of risk factors and regular monitoring to address issues raised by patients and caregivers.

## Ethical approval

The ethical approval was obtained from a joint decision of the Scientific Council of the Faculty of Health Sciences of Zinder University and the Advisory Technical Board of Zinder National Hospital, Ref.: FSS-UZ/HNZ-CTC-0014-02-05-2017.

## Sources of funding

None.

## Author contribution

Harissou Adamou, Ibrahim Amadou Magagi, Ousseini Adakal: have conceived and designed the study, written and drafted the manuscript.

Mahamadou Doutchi, Oumarou Habou, Mamane Boukari, Lassey James Didier, Rachid Sani: have all contributed to the management, writing. All authors in this manuscript contributed to drafting and writing of this manuscript and approved the final manuscript.

## Research registration Unique Identifying number (UIN)

Name of the registry: **Research Registry**
Unique Identifying number or registration ID: **researchregistry5224**
Hyperlink to the registration (must be publicly accessible): https://www.researchregistry.com/browse-the-registry#home/

## Guarantor

Harissou Adamou.

## Declaration of competing interest

None.
